# Integrative Analysis of Longitudinal Metabolomics Data from a Personal Multi-Omics Profile 

**DOI:** 10.3390/metabo3030741

**Published:** 2013-09-03

**Authors:** Larissa Stanberry, George I. Mias, Winston Haynes, Roger Higdon, Michael Snyder, Eugene Kolker

**Affiliations:** 1 Bioinformatics and High-throughput Analysis Laboratory, and High-throughput Analysis Core, Seattle Children’s Research Institute, Seattle, 98101, USA; E-Mails: winston.haynes@seattlechildrens.org (W.H.); roger.higdon@seattlechildrens.org (R.H.); eugene.kolker@seattlechildrens.org (E.K.); 2 Predictive Analytics, Seattle Children’s, Seattle, 98101, USA; 3 Data-Enabled Life Sciences Alliance (DELSA Global), Seattle, 98101, USA; E-Mail: mpsnyder@stanford.edu (M.S.); 4 Department of Genetics, Stanford University School of Medicine, Palo Alto, CA, 94305, USA; E-Mail: george.mias@stanford.edu (G.I.M.); 5 Stanford Center for Genomics and Personalized Medicine, Palo Alto, CA, 94305, USA; 6 Departments of Biomedical Informatics & Medical Education and Pediatrics, University of Washington, Seattle, WA, 98195, USA

**Keywords:** metabolomics, integrative pathway analysis, DEAP, dendrogram sharpening, DELSA, iPOP, longitudinal design, multi-omics data, single linkage

## Abstract

The integrative personal omics profile (iPOP) is a pioneering study that combines genomics, transcriptomics, proteomics, metabolomics and autoantibody profiles from a single individual over a 14-month period. The observation period includes two episodes of viral infection: a human rhinovirus and a respiratory syncytial virus. The profile studies give an informative snapshot into the biological functioning of an organism. We hypothesize that pathway expression levels are associated with disease status. To test this hypothesis, we use biological pathways to integrate metabolomics and proteomics iPOP data. The approach computes the pathways’ differential expression levels at each time point, while taking into account the pathway structure and the longitudinal design. The resulting pathway levels show strong association with the disease status. Further, we identify temporal patterns in metabolite expression levels. The changes in metabolite expression levels also appear to be consistent with the disease status. The results of the integrative analysis suggest that changes in biological pathways may be used to predict and monitor the disease. The iPOP experimental design, data acquisition and analysis issues are discussed within the broader context of personal profiling.

## 1. Introduction

Modern high-throughput technologies enable rapid and efficient simultaneous acquisition of multi-omics data in the course of a single experiment. The combination of genomics, transcriptomics, proteomics, lipidomics and metabolomics data provides a snapshot of biological processes in an organism. As such, multi-omics studies are essential to advance the knowledge of biological systems [1,2], understand, predict, diagnose and monitor diseases [1,2,3], discover biomarkers [4] and identify drug targets [3]. 

Compared to single omics approaches, multi-omics data provide a comprehensive view of biochemical, biophysical, genetic and epigenetic processes in an organism. However, data vary considerably between each different omics, not only with respect to the biological processes the data represent, but also the associated noise levels, identification accuracy, coverage and temporal resolution of the data. These differences complicate integration and joint modeling of multi-omics data. 

Effectively identifying underlying factors and estimating their effects on the system requires advanced analysis tools capable of integrating multi-omics data. Currently, multi-omics studies rarely utilize integrative approaches. Instead, each omics dataset is analyzed separately, and the outcomes are merged together for joint interpretation. A number of integrative multi-omics analysis approaches were recently proposed, including: iCluster [5], PARADIGM (Pathway Recognition Algorithm using Data Integration on Genomic Models) [6] and factor analysis [1]. Both iCluster and factor analysis use a latent-variable approach to identify grouping structure in the data. In contrast, PARADIGM uses integrated omics data to infer the pathways activities. 

Integrated multi-omics studies are becoming increasingly important in the context of personalized medicine, where treatment decisions are based on patients’ omics, demographic, clinical and environmental data [7,8,9,10,11]. The recently released integrative personal omics profile (iPOP) study is a pioneering work in the field of personalized omics profiling. The study sampled genomics, transcriptomics, proteomics, metabolomics and autoantibody profiles of a single individual (Dr. Michael Snyder) over a 14-month period. The study revealed a number of medical risks, dynamic changes in multi-omics components over time and an association between the multi-omics expressions and disease status [12]. 

The iPOP study revealed the potential merits and advantages over conventional clinical methods of extensive multi-omics profiling in a patient for monitoring, forecasting and diagnosing. As the first in-depth investigation, the iPOP experiment highlighted the importance of a comprehensive experimental design and the necessity of advanced analytic tools applicable to large-scale multi-omics data. The iPOP study collected more than 30 TB of data. Storage, annotation, analysis and sharing of these data requires an array of skills and expertise, large compute power, access to a variety of resources and databases, pre-determined formats, sophisticated software, advanced analytic tools and visualization capabilities [13,14,15,16,17,18,19,20,21]. The scope of the iPOP study and the breadth of the acquired data are unparalleled in their complexity and richness. As such, the iPOP represents a unique and extensive resource for multi-omics integration, clinical application of personalized profiling, tool development, data formatting and sharing. 

Due to its novelty, uniqueness and diversity, the iPOP study was selected as one of the two landmark experiments in the Quantified Human Initiative launched by DELSA Global (Data-Enabled Life Sciences Alliance; delsaglobal.org) [22]. The goal of the initiative is to model the underlying biological dynamics of the human organism on the micro-and macro-scales through collective innovation [22,23,24]. 

This study is focused primarily on the metabolomics data from the iPOP study. Metabolomics data are a principal component of the multi-omics profiling. As compared to genomics and transcriptomics data, metabolomics is a dynamic reflection of the functional state of an organism, as well as environmental factors [25]. Understanding the diversity of and changes in metabolomics data and their interactions with other omics is essential to advancing personalized diagnostics and medicine. 

In the iPOP study, the observation period included two episodes of viral infection: a human rhinovirus (HRV) and a respiratory syncytial virus (RSV). We hypothesize that pathway expression levels are associated with the disease status. To test this hypothesis, we use biological pathways as a primary model to integrate metabolomics and proteomics data [26]. From multi-omics expression data, we compute the pathways’ differential expression levels over time. The resulting pathway scores take into account expression data, pathway structure and the longitudinal design of the study. We also implement an enhanced unsupervised clustering technique to identify groups of metabolites exhibiting coherent temporal changes. 

In what follows, we provide a brief overview of the data and methods. We then give a detailed description of the results. We discuss the patterns and dependencies identified in the data, the merits of the applied analysis methods, the value of the multi-omics personal profiling and the benefits and challenges of the longitudinal multi-omics studies. We conclude with a summary of the results and a list of recommendations and open questions. 

## 2. Data and Methods

### 2.1. Data Collection and Pre-Processing 

**Figure 1:**
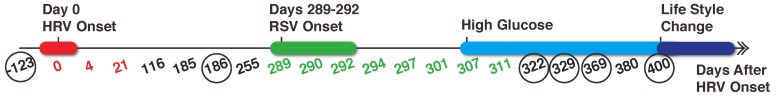
The timeline of the study. The subject was monitored for 726 days. Days of human rhinovirus (HRV) and respiratory syncytial virus (RSV) infections are marked in red and green, respectively. The red and green bars represent the onset of the infections. The light blue bar shows the period of high glucose levels, and the dark blue one indicates lifestyle changes, including (1) increased exercise, (2) ingestion of 81 mg of acetylsalicylic acid and ibuprofen each day (the latter only during the first six weeks of this period) and (3) substantially reduced sugar intake. Circled days indicate fasted time points.

Tissue samples were collected, processed and analyzed using metabolomics and proteomics methods, as detailed in [12]. Detailed phenotypic data can also be found in [12]. Figure 1 shows the timeline of the study. During the observation period, the subject experienced two viral infections: a human rhinovirus (HRV) and a respiratory syncytial virus (RSV). The onsets of the infections are labeled as day 0 and 289, respectively. 

### 2.2. Metabolomics Data 

Metabolome data were acquired throughout the study, as marked in Figure 1 with the exception of days 21, 186, 329 and 400. The samples were run in two batches. The first batch included days -123, 0, 4, 21, 116 and 185, and the second batch included samples from days 255 onward. 

During the HRV infection, metabolites were measured twice (days 0 and 4). Consequently, 7,361 distinct serum metabolite m/z intensities were measured at least once and 7,019 were measured consistently over time. During the RSV infection (days 255–400), metabolomics data were collected eight times with 5,131 distinct serum metabolite m/z intensities tracked consistently, including 4,217 observed at every time instance and 1,098 measured in the HRV period. Further, we extracted ChEBI identifiers for 198 of these metabolic compounds [27]. 

### 2.3. Proteomics Data 

Relative expression levels of serum proteins were acquired for days 0, 4 and 21. The levels were calculated relative to day 116. Overall, 664 proteins were consistently quantified, and their UniProt identifiers were recovered [28]. 

For the peripheral blood mononuclear cell (PBMC) proteome, spectra were obtained from three Tandem Mass Tag-labeled samples with three technical replicates each. The data were collected from day 186 onward, except for day 329. Relative expressions were calculated with respect to a healthy time point at day 255. Within each sample, the ratios were normalized to have a unit mean. For quality control and reproducibility assessment, the 131/126 intensity ratio was replicated for 126 and 131 amumass tags corresponding to days 255 and 301. The replicated ratio was averaged across samples and rescaled to have a unit mean. Overall relative expression levels for 7,041 proteins were quantified, out of which 3,066 were observed consistently across the 14 time points. For more details on data acquisition and pre-processing, see [12]. 

### 2.4. Cluster Analysis 

We applied agglomerative hierarchical single linkage clustering (SLC) to identify clusters of compounds with similar temporal profiles. The dissimilarity between two compounds, *x* and *y*, is given by 1 − *ρ_xy_*, where *ρ_xy_* is the correlation coefficient between the time courses of the corresponding compounds. The hierarchy is then built from the bottom up, *i.e*., each compound initially represents a cluster and, at each step, the two closest clusters are merged together into a binary tree, also called a dendrogram. In SLC, the distance between two clusters is defined as the minimum of all pairwise distances between points in the clusters [29,30]. 

The single linkage was shown to be fractionally consistent, *i.e*., in the presence of two disjoint population groups, there will be two distinct single linkage clusters containing a positive fraction of the sample points from the corresponding groups. Hence, the single linkage is conservative, in the sense that it will identify sufficiently separated modal regions [31]. In turn, complete and average linkage methods produce accurate clustering only for data constituted of well-separated groups. In the presence of noise, the complete and average linkage methods fail to correctly identify the grouping structure. It has been shown that the clusters produced by these algorithms depend on the range, but not on the density, of data [29]. 

**Figure 2:**
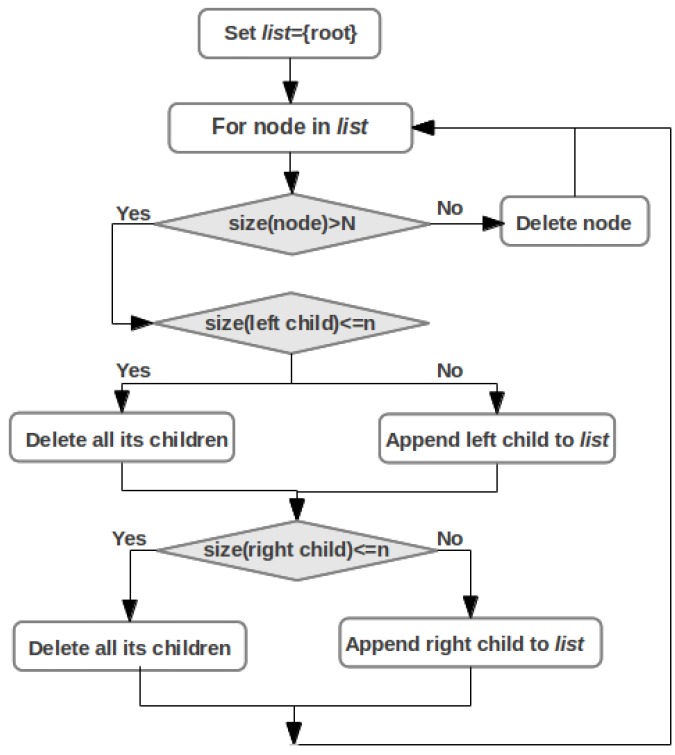
The dendrogram sharpening algorithm.

In the presence of noise, SLC exhibits a chaining effect, with dendrogram top nodes having one very small and one very large child. Although the chaining correctly indicates the lack of spatial separation between clusters, it hinders the grouping structure. Sharpening methods allow for effectively reducing the size of data and making the structure more apparent [32]. The dendrogram sharpening prunes the linkage tree to reduce noise and increase the contrast between the modal regions. More specifically, child nodes of a size less than *n* are discarded, if the size of the corresponding parent node exceeds *N*. The recursive algorithm is applied directly to the tree and is simple to implement (see Figure 2). The sharpening uses two parameters, *n* and *N*. Parameter *N* defines whether or not the subtree of a given node should be filtered. For high noise levels, multiple passes of the algorithm are advantageous. 

In the sharpened tree, the cluster cores are identified recursively, starting from the root node. Conventional methods threshold the tree at a pre-specified global cut-off value. In contrast, to identify cluster groups, we compute a robust statistic using the distribution of similarity measures within each qualifying subtree. More specifically, the left child is defined to be a cluster if the distance between the left and right children exceeds *M* + *IQR*, where *M* and *IQR* are the median and interquartile range of the left subtree. Similarly, for the right child. The single-linkage clustering with dendrogram sharpening was shown to correctly identify the multimodal structure of the data in the simulation studies and was applied to neuroimaging and gene expression data [33,34]. 

Here, we applied the dendrogram sharpening twice. The first filtering with (*n*, *N*) = (2, 30) effectively eliminates isolated data points. The second iteration with (*n*, *N*) = (10, 30) filters relatively small clusters spuriously formed in the sharpened data. 

Data filtered in sharpening are reassigned to the identified cluster cores. For example, one can reassign filtered data using the nearest neighbor approach. Here, we used the nearest centroid to classify the filtered values. For that, for each identified cluster, we compute its mean time course. We then calculate pairwise correlations between the mean time course and filtered data. The filtered data are assigned to the cluster with the highest correlation, provided the correlation coefficient is above a certain threshold. This avoids contaminating clusters with noisy observations, hence preserving the quality of the signal. Here, we used the threshold of 0.5 to reassigned filtered data. This threshold is considerably lower than the intracluster correlation. This ensures an increase in cluster heterogeneity, while preserving the signal quality. 

### 2.5. Pathway Mapping 

We employed Reactome to map metabolites and proteins to pathways [17]. Reactome stores information on biological pathways, including the proteins, metabolites and their regulation patterns. To extract the information on structure and content of metabolic pathways, we used a simplified pathway file format, where each line represents an individual reactions in a pathway. The file format enables easy parsing and manipulation [21]. A full database dump was downloaded from Reactome, version April 5, 2013 [17]. Using Protege, we determined the database representations of the pathway information [35]. From Reactome, we identified inputs, regulators and outputs of each reaction. The Reactome database was processed using a combination of custom Python and SQLscripts. For metabolites and proteins, we extracted ChEBI and UniProt identifiers, respectively [27,28]. 

### 2.6. Integrative Pathway Analysis 

To integrate metabolomics and proteomics data, we extended the Differential Expression Analysis for Pathways (DEAP) to include multi-omics measurements and to account for the longitudinal design. In DEAP, each pathway is viewed as a union of distinct subpaths. The representation is not unique, and the subpaths are not mutually exclusive. The score for each identified subpath is computed as a weighted sum of the expression levels of its components, where weights are either +1 or −1 when the reaction is catalytic or inhibitory, respectively. The DEAP pathway score is then given by the maximum score of the constituting subpaths. Consequently, the DEAP score takes into account the structure of the pathway and was shown to have more sensitivity and power as compared to other pathway statistics and analysis approaches. For more details, see [21]. 

To account for the longitudinal design, for each identified compound, we used the following algorithm.

Let *i* index pathways, let *t* = 1,...,*T* index the time points and let *j* = 1,..., *N_i_* index the molecules in pathway *i*. Denote by *e* a log-relative expression of molecular compounds.

For each pathway, *i*, compute temporal scores, *λ_it_*, as follows:

1. For each molecule, *j*, in pathway *i*, calculate the average log-relative expression, *m_ij_*, across the time points: 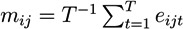.
2. From *m_ij_*, *j* = 1,..., *N_i_*, use DEAP to identify the maximally scoring subpath and its constituting components: *S_i_* = {*j*_1_,..., *j_M_*_(*i*)_}.
3. Given *S_i_*, at each time point, compute the score, 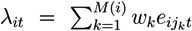, where weights, *w_k_*, correspond to +1 and −1, respectively.

For each pathway, this algorithm produces a series of scores, {*λ_i_*_1_,..., *λ_iT_*}. The scores take into account both the relative expressions of the constituting compounds, as well as the pathway structure. As such, they can be thought of as proxy measures of pathway expression levels over time. 

## 3. Results

### 3.1. Cluster Analysis 

**Figure 3:**
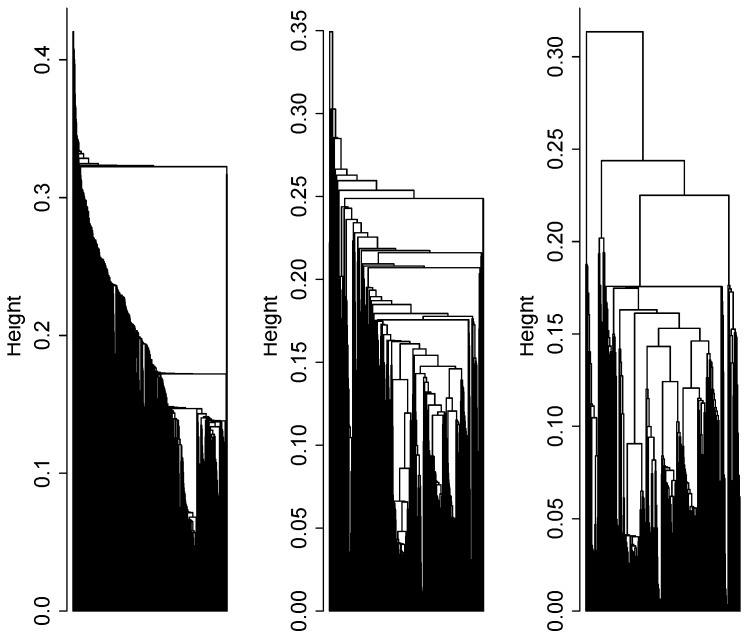
(Left to right) dendrogram trees for the full, once- and twice-sharpened data containing 1,098, 545 and 293 complete data points on metabolic compounds.

We applied dendrogram sharpening to the complete metabolomics data set containing 1,088 compounds. Figure 3 demonstrates how the data structure becomes more pronounced with each path of the sharpening algorithm. From the twice-sharpened data, we identified eight metabolic cluster cores containing 136 metabolites. The similarity of the individual time courses within each cluster core was of note, with mean/median correlation coefficient of 0.87/0.91. After reassigning filtered data, the expanded clusters contained 724 metabolites. 

**Figure 4:**
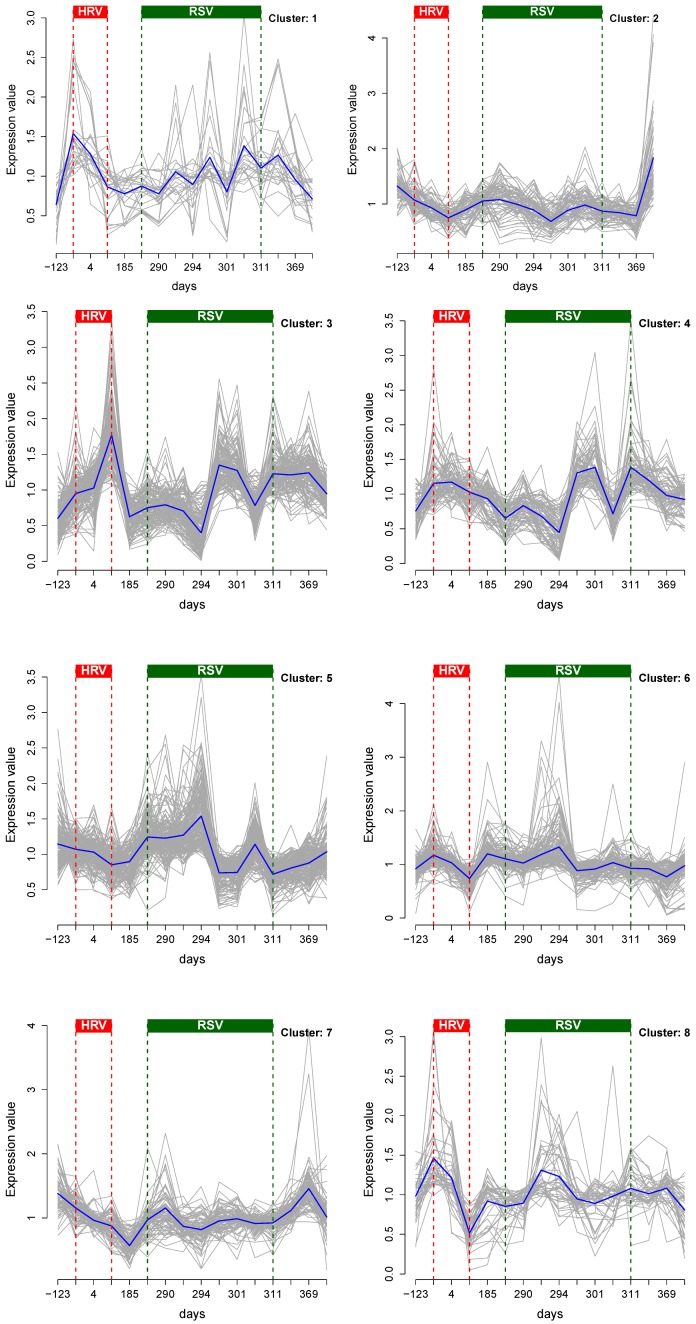
Eight distinct clusters of the metabolome profiles in the personal omics profile (iPOP) study showing individual (grey) and mean (blue) metabolite time courses for each cluster. Also marked are periods of HRV and RSV infections.

The clusters showed distinct temporal patterns (Figure 4). For example, expression levels for metabolites in Cluster 3 are suppressed at the onset of both HRV and RSV, but elevated at the end of the infection. Clusters 5 and 6 represent metabolites whose levels are elevated at the beginning of the infection, but return to (presumably) basal levels as the subject recovers. Clusters 3 and 4 also show a brief increase in expression levels at the end of RSV. 

The features of the metabolite patterns during the HRV infection are less pronounced, due to a lack of data acquired in that period. However, the patterns show the discordance of expression levels at the end of the infection relative to the onset; see Clusters 3, 4–8 (Figure 4). The identified metabolites were implicated in estrogen and androgen metabolism, metabolism of lipids and lipoproteins, bile secretion, steroid hormone biosynthesis, biosynthesis of unsaturated fatty acids, purine and lysine metabolism and urate biosynthesis pathways. The networks of over-represented pathways in Clusters 2, 3, 6, 7 and 8 are shown in the supplementary figure. The identified metabolite clusters are not pathway-specific. The pathway mapping and over-representation analysis was done using ConsensusPathDB [33,36] 

### 3.2. Integrative Pathway Analysis 

For the integrated pathway analysis, the iPOP PBMC and serum proteomics and serum metabolomics data were mapped to pathways, as described earlier. For serum metabolomics, relative expressions were computed for each time point relative to a healthy time point at day 255. For the RSV period, 877 pathways contained only proteins, eight contained proteins and metabolites and eight contained only metabolites. For the HRV period, the corresponding numbers were 376, six and 11. 

**Figure 5:**
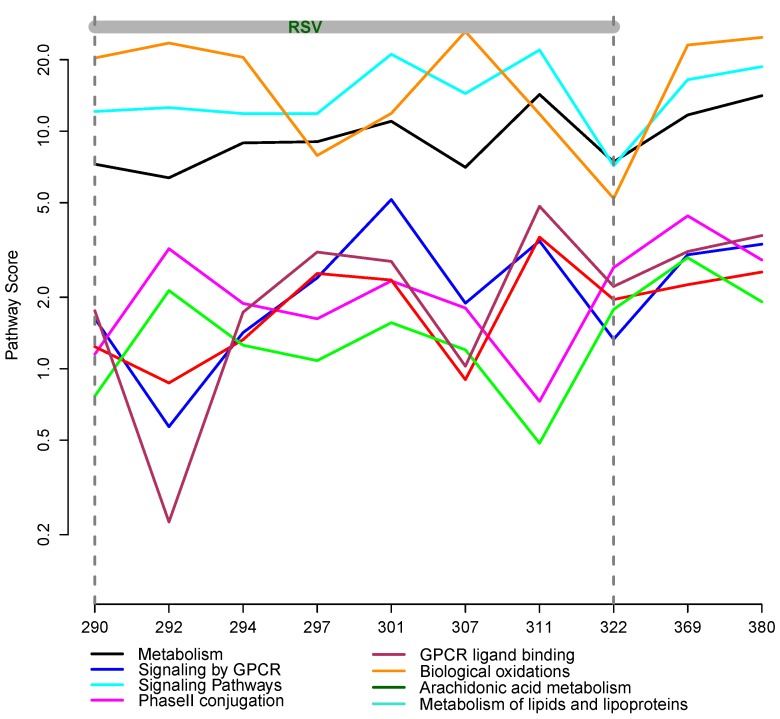
Changes in functional pathway scores over time. Each pathway contained both measured metabolites and proteins. The pathways are color-coded according to the legend.

Figure 5 shows the trajectories of the pathway scores for the eight overlapping pathways containing serum metabolites and PBMC proteins. There appears to be an increase in pathway scores over time for the metabolism (black) and GPCRligand-binding (maroon) pathways. 

**Figure 6:**
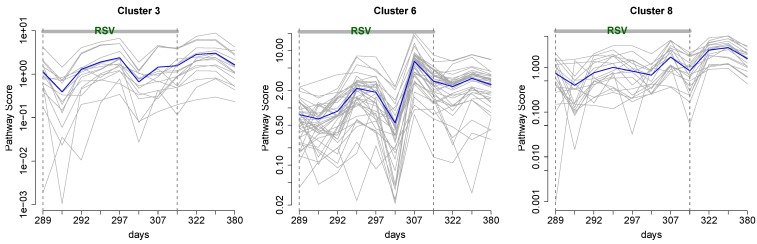
Three out of eight clusters showing distinct temporal patterns of the pathway scores for serum metabolome and PBMC proteome. Individual pathway scores are shown in grey; mean scores are in blue.

To explore the pathway patterns, we considered 533 metabolic pathways with unique scores that contained serum metabolic and/or PBMC proteomic compounds. The pathway scores were computed for each time point. Applying the clustering algorithm, we identified eight clusters, three of which are shown in Figure 6. The changes in the pathway scores over time appear to correlate with the disease status. In all three clusters, the scores increase in magnitude as the disease progresses. 

Cluster 3 contains 16 pathways, including virus assembly and release, SMAC-mediated apoptosis response, NEF (Negative Regulatory Factor) and signal transduction, collagen biosynthesis and modifying enzymes and insulin processing. Cluster 6 contains 39 pathways, including TLR3 and TLR4 (Toll Like Receptor) cascades, Interleukin 2 (IL2) signaling, SOS-mediated signaling, signaling by EGFR and FGFR (epidermal and fibroblast growth factor receptors, respectively) in disease and ERBB4 signaling. Cluster 8 contains 19 pathways, including mTOR (mammalian target of rapamycin) signaling, TCR (T-cell receptor) signaling, generation of second messenger molecules, fibronectin matrix formation and interferon gamma signaling. 

Many of the identified pathways are known to be activated in response to viral infection. In particular, previous studies showed signaling receptors, TLRs and IL2, to be the important components in the immune response to RSV. Both IL2 and TLR4 are known to contribute to cytokine secretion and activation, respectively [37,38,39]. FGFR is involved in mucin production, while both EGFR and FGFR stimulate differentiation and proliferation of cells, processes that are crucial in tissue repair and response to injury [40,41]. EGFR activation by RSV was shown to be associated with inflammation and cell survival [42]. mTOR serves as a central regulator for cellular metabolism, growth and survival [43]. Further, SMAC and NEF are associated with apoptosis. 

Pathway trajectories in Cluster 6 spike at days 301 and 307. The spike at 301 was also observed in cytokines levels [12]. The spikes in pathway scores may be associated with medication ingestion, possibly anti-inflammatory drugs. Furthermore, from the mean time course in Cluster 3, we observed that an upward trend in the pathway score started after day 301, coinciding with the beginning of the elevated glucose levels in the subject (see [12], Figure 2D). A steady increase in pathway scores could be attributed to the disease etiology, as the virus may be present for as long as six weeks after the infection occurs. 

## 4. Discussion

Personal multi-omics profiles are poised to become instrumental in personalized medicine [12,44,45,46,47,48,49,50,51]. The iPOP study pioneered extensive personal multi-omics profiling as a monitoring and predicting tool. The study collected a large volume of rich and complex data that contain information on biological processes, organism functioning and environmental effects. These multi-omics data can be leveraged to identify medical risks, dynamic changes in omics components over time and the association between expression levels and disease status. 

Currently, genetic testing is widely used to identify genetic disease risk or to diagnose a genetic condition. More recently, peripheral blood gene expression data were shown to correctly discriminate between HRV, RSV or influenza A and bacterial infection [52]. Another study showed that miR-451 was differentially expressed in influenza infection [53]. 

In this work, we used an integrative analysis approach to identify distinct temporal patterns in pathway and metabolite differential expression levels. The temporal changes appear to be associated with the disease status. Due to a lack of measures of disease severity and progression, we were not able to establish the strength of the association. Nevertheless, these findings suggest that changes in pathway expression levels may be used to predict the disease, identify disease etiology and differentiate responses to medication. This may be accomplished through deeper integration of genomics, transcriptomics, metabolomics and proteomics data, together with clinical measurements and environmental variables. Integrating these diverse data would enable comprehensive modeling of the interactions between the biological components and systems. 

The iPOP study not only opened new horizons for multi-omics application in personalized medicine and medical sciences, but also highlighted the complexities of personal omics profiling when it comes to design, data collection and analysis. As a newly emerging trend in data-enabled life sciences [54], more quantified self multi-omics studies are certain to come out. To enhance the quality of future research and to drive methodological and experimental developments, we discuss the main findings, the applicability of the proposed analysis methods and the challenges and advantages of longitudinal iPOP studies. 

**Study Design.** The study was observational in nature and spanned a period of nearly 14 months. The longitudinal design allowed the collection of a large volume of data under a variety of conditions, thus enabling investigation of the dynamics of omics expressions. As such, the data contain unprecedented insight and represent a detailed view into the functioning of the organism. On the analytic side, the longitudinal acquisition considerably increased the volume of data to be analyzed and introduced a temporal component that is absent in a typical cross-sectional omics study. 

During the study, the subject had two different viral infections. Both infection periods were approximately 20 days long; however, the metabolomics data were acquired twice during the HRV infection and eight times during the RSV infection. The higher rate of data acquisition during the RSV enabled identification of distinct temporal changes in metabolic profiles as the infection progressed. The sparse coverage during the HRV infection only allowed very general observations. 

From the design perspective, the infection periods demonstrate the difficulty of maintaining the consistency of data sampling throughout the course of the study. In the iPOP study, investigators were aiming to acquire as comprehensive a data set as possible within a certain time frame. In future personal omics profiles, the design and planning should be tailored to the goal of the study. For example, the goal of profiling an individual in a healthy state would imply that acquisition during the infection periods should be avoided or excluded if collected. Another important issue is to correctly estimate the onset of the disease, as biological changes may happen before symptoms become apparent. 

Longitudinal studies should also maintain consistent tissue sampling and processing protocols. This is important from the design perspective and especially relevant to metabolomics studies, where short-term effects of exogenous factors are more likely to occur. In the iPOP study, fasting did not seem to affect the number or the expression values of the identified compounds. However, this cannot be definitively ascertained, given the small number of fasting samples. 

During the course of the study, the subject underwent a number of lifestyle and dietary changes, including increased exercise, medication uptake and reduced sugar intake. Given the dynamic nature of metabolomics, life-style factors are most likely to impact the metabolome profiling. Hence, it is prudent to maintain detailed records throughout the study, including basic physiological measurements and a wellness diary. In practice, acquiring regular detailed assessment without overwhelming the participant is challenging. A standardized daily survey may be an effective way to collect the relevant information. 

The volume and complexity of the data implies that care should be taken when acquiring and pre-processing the data. For the iPOP metabolome, tissue samples were run in two batches. The number of metabolites identified in the first batch was larger by about 2,000 compounds, as compared to the second batch. Since the samples were grouped sequentially, it cannot be reliably determined whether the difference in the number of identifications is due to the technical component (sample preparation and instrumental run) or a subject factor (e.g., lifestyle changes). In addition, the infection factor was also confounded with the batch number. These uncertainties underscore the importance of randomization during sample preparation, instrumental analysis and data pre-processing. 

**Cluster Analysis.** The longitudinal component of the study drastically increased the amount of data acquired for the analysis. We utilized clustering methods to study the patterns of temporal trajectories in metabolomics data and their association with the disease status. 

In the original paper, the authors grouped the data into three classes, categorized as the spike maxima, spike minima and autocorrelation class, which were subsequently clustered within each class. However, because the data were acquired over time on the same subject, we would expect them to exhibit a certain degree of autocorrelation. Hence, the original paper analysis included removal of autocorrelated components prior to spike maxima/minima classification, to minimize spike class overlaps with the autocorrelation class. The SLC used here was shown to correctly identify sufficiently separated modal regions [29]. The dendrogram sharpening removes noisy observations and makes the data structure more apparent. As such, SLC with dendrogram sharpening is well-suited for the analysis of large, noisy data [33]. 

The identified temporal patterns in metabolic profiles indicate that the metabolite expression may correlate with the disease status. The metabolite expression profiles appear to fall into two categories: elevated at the onset and slowly declining during disease progression and the inverse. Based on the available data, we cannot determine whether the observed metabolic changes over time are specific to particular medication and/or dietary interventions and/or biological response to the infection. Similarly, since the metabolomics data were sampled considerably less frequently during the HRV infection as compared to RSV, it cannot be reliably determined whether the observed patterns are specific to the type of the infection. 

**Integrative Omics Analysis.** In this study, we also applied a pathway-centric integration approach to multi-omics data analysis [26]. Frequently, different omics acquired in a single experiment are analyzed separately, and the results of the omic-specific analyses are then interpreted in some integrated fashion [55,56]. However, integrative analysis methods are more advantageous than single omics methods, as the acquired omics data are driven by the same underlying biological mechanisms; therefore, integrating the interdependent omics data increases the statistical power of the analysis and the accuracy of the model estimates. Hence, taken together, multi-omics data provide a more comprehensive view of biological processes. 

Current integrative approaches are discriminative in nature, as they attempt to classify the data into groups based on multi-omics observations [1,5]. These approaches are not applicable to the longitudinal multi-omics studies, like the iPOP study. In this paper, we introduce a pathway-centric integrative approach to analyze longitudinal multi-omics data. The method is an extension of the recently developed DEAP analysis [21]. The method effectively combines metabolite and protein expression data, while taking into account the pathway structure and longitudinal design. The resulting score series reflect the function of a given pathway at each time point. The scores can be interpreted separately for each pathway or analyzed jointly to identify pathway groups exhibiting similar temporal patterns. Currently, the method integrates metabolomics and proteomics data at each time point separately. The method could be extended and enhanced to include gene expression data, the temporal dependence and covariates. This alteration would require a higher data sampling rate. 

From the integrative analysis of the iPOP proteomics and metabolomics data, differential expression levels of pathways appear to correlate with disease state. This suggests that integrative multi-omics pathway functional scores reflect the ongoing biological processes in the organism. These early results provide evidence for the advantages of multi-omics data integration. 

**Missing Data.** The missing data problem is ubiquitous in high-throughput experiments. A number of methods for data imputation have been proposed in the literature [57,58,59]. The iPOP study represents a particular challenge with its longitudinal layout, an array of explanatory variables and confounding factors. Given the complexity of the design and data, imputing missing data would have introduced sizable error. Hence, we abstained from imputing or otherwise inferring the missing data. The missing data problem in the context of high-throughput personalized multi-omics studies merits careful investigation that was beyond the scope of this paper. 

## 5. Conclusions

We have performed a detailed integrative analysis of the metabolomics and proteomics data acquired in the pioneering iPOP study. In this study, we used a pathway-centric approach to integrate the metabolomics and proteomics data, while taking into accoung the longitudinal design of the study. From integrated data, we identified temporal patterns in pathway expression levels that were consistent with disease status and progression. The iPOP study showcased both the promise and challenge of the personal multi-omics profile studies. Based on our analysis, we discussed specific recommendations for multi-omics profile studies, including consistent protocols for data acquisition, the need for randomization in sample preparation and instrumental data collection and the choice of appropriate analysis tools. 

## 6. Data Dissemination

The location and details of the raw data repository for the iPOP experiment is described in [12]. Pre-processed metabolomics and proteomics data, mapped pathways and other supplementary material currently accessible through the complete list of the pathways in each cluster will be made available at , Dryad Digital Repository. In the near future, metabolomics and proteomics data will be accessible through MOPEDunder the experiment name, snyder_personal_omics_-profiling [60]. 

## Figures and Tables

**Figure S1:**
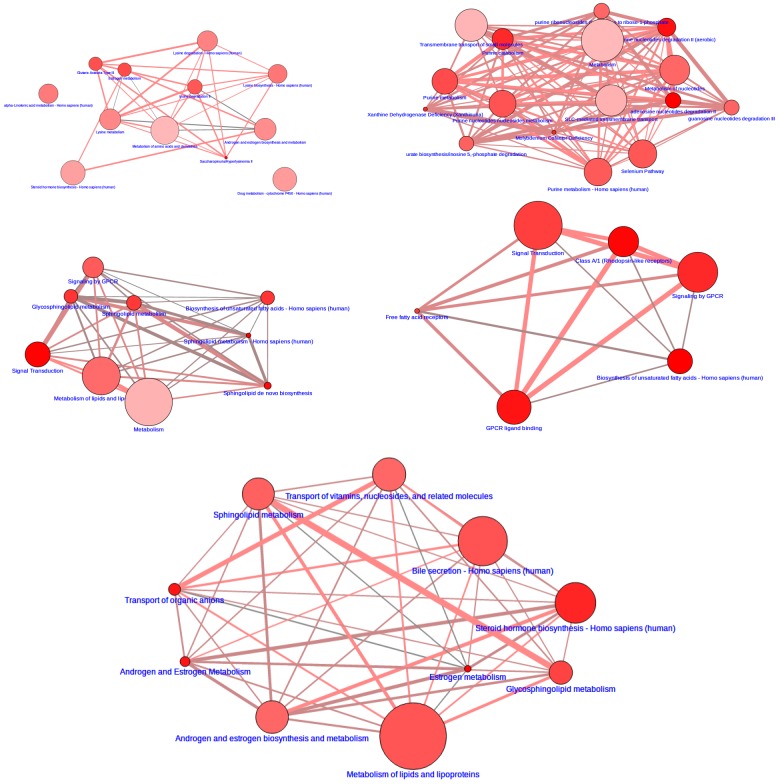
Pathway networks for Clusters 2,3,6,7,8 (counterclockwise from the left top corner). The node size reflects the total number of components in a pathway; the node color reflects the *p*-value of the pathway representation analysis (a darker color corresponds to lower *p*-values); the edge width corresponds to a relative number of shared compounds between the pathways; and the edge color reflects the absolute number in the overlap.

## References

[1] Liu Y., Devescovi V., Chen S., Nardini C. (2013). Multilevel omic data integration in cancer cell lines: Advanced annotation and emergent properties. BMC Syst. Biol..

[2] Liu Q., Halvey P.J., Shyr Y., Slebos R.J.C., Liebler D.C., Zhang B. (2013). Integrative omics analysis reveals the importance and scope of translational repression in microRNA-mediated regulation. Mol. Cell. Proteomics: MCP.

[3] Kurland I.J., Accili D., Burant C., Fischer S.M., Kahn B.B., Newgard C.B., Ramagiri S., Ronnett G.V., Ryals J.A., Sanders M. (2013). Application of combined omics platforms to accelerate biomedical discovery in diabesity. Ann. N.Y. Acad. Sci..

[4] Blanchet L., Smolinska A., Attali A., Stoop M.P., Ampt K.A.M., van Aken H., Suidgeest E., Tuinstra T., Wijmenga S.S. (2011). Fusion of metabolomics and proteomics data for biomarkers discovery: Case study on the experimental autoimmune encephalomyelitis. BMC Bioinforma..

[5] Shen R., Olshen A.B., Ladanyi M. (2009). Integrative clustering of multiple genomic data types using a joint latent variable model with application to breast and lung cancer subtype analysis. Bioinformatics.

[6] Vaske C.J., Benz S.C., Sanborn J.Z., Earl D., Szeto C., Zhu J., Haussler D., Stuart J.M. (2010). Inference of patient-specific pathway activities from multi-dimensional cancer genomics data using PARADIGM. Bioinformatics.

[7] Vignot S., Soria J.C. (2013). Discrepancies between primary tumor and metastasis: Impact on personalized medicine. Bull. Cancer.

[8] Law G.L., Korth M.J., Benecke A.G., Katze M.G. (2013). Systems virology: Host-directed approaches to viral pathogenesis and drug targeting. Nat. Rev. Microbiol..

[9] Tanrıkulu A., Ağırbaşlı M. (2013). Triple therapy (aspirin, clopidogrel and oral anticoagulant) after percutaneous coronary intervention: another call for personalized medicine. Anadolu Kardiyol Derg..

[10] Blackwell L.S., Marciel K.K., Quittner A.L. (2013). Utilization of patient-reported outcomes as a step towards collaborative medicine. Paediatr. Respir. Rev..

[11] Buyse M., Michiels S. (2013). Omics-based clinical trial designs. Curr. Opin. Oncol..

[12] Chen R., Mias G.I., Li-Pook-Than J., Jiang L., Lam H.Y.K., Chen R., Miriami E., Karczewski K.J., Hariharan M., Dewey F.E. (2012). Personal omics profiling reveals dynamic molecular and medical phenotypes. Cell.

[13] Higdon R., Haynes W., Stanberry L., Stewart E., Yandl G., Howard C., Broomall W., Kolker N., Kolker E. (2013). Unraveling the complexities of life sciences data. Big Data.

[14] Kolker E., Stewart E., zdemir V. (2012). DELSA global for “Big Data” and the Bioeconomy: Catalyzing Collective Innovation. Ind. Biotechnol..

[15] Kolker E. (2011). Editorial: Special issue on data-intensive science. OMICS.

[16] Barga R., Howe B., Beck D., Bowers S., Dobyns W., Haynes W., Higdon R., Howard C., Roth C., Stewart E. (2011). Bioinformatics and data-intensive scientific discovery in the beginning of the 21st century. Omics: A J. Integr. Biol..

[17] Matthews L., Gopinath G., Gillespie M., Caudy M., Croft D., de Bono B., Garapati P., Hemish J., Hermjakob H., Jassal B. (2009). Reactome knowledgebase of human biological pathways and processes. Nucleic Acids Res..

[18] Ogata H., Goto S., Sato K., Fujibuchi W., Bono H., Kanehisa M. (1999). KEGG: Kyoto encyclopedia of genes and genomes. Nucleic Acids Res..

[19] Fox G., Qiu X., Beason S., Choi J.Y., Ekanayake J., Gunarathne T., Rho M., Tang H., Devadasan N., Liu G. (2009). Biomedical Case Studies in Data Intensive Computing. Proceedings of the CloudCom ’09 Proceedings of the 1st International Conference on Cloud Computing.

[20] Gilbert D.R., Schroeder M., van Helden J. (2000). Interactive visualization and exploration of relationships between biological objects. Trends Biotechnol..

[21] Haynes W.A., Higdon R., Stanberry L., Collins D., Kolker E. (2013). Differential expression analysis for pathways. PLoS Comput. Biol..

[22] Kolker E., Higdon R., Welch D., Bauman A., Stewart E., Haynes W., Broomall W., Kolker N. (2012). Corrigendum to “SPIRE: Systematic protein investigative research environment” [J. Proteomics 75 (1) (2011) 122–126]. J. Proteomics.

[23] Ozdemir V., Pang T., Knoppers B.M., Avard D., Faraj S.A., Zawati M.H., Kolker E. (2011). Vaccines of the 21st century and vaccinomics: Data-enabled science meets global health to spark collective action for vaccine innovation. OMICS: A J. Integr. Biol..

[24] Stewart E., Kolker E. (2013). DELSA global workshop: Quantified human initiative. Big Data.

[25] Ryan D., Robards K. (2006). Metabolomics: The greatest omics of them all?. Anal. Chem..

[26] Stanberry L., Haynes W., Higdon R., Kolker E. Pathway-centric analysis for multi-omics data.

[27] Hastings J., de Matos P., Dekker A., Ennis M., Harsha B., Kale N., Muthukrishnan V., Owen G., Turner S., Williams M. (2013). The ChEBI reference database and ontology for biologically relevant chemistry: Enhancements for 2013. Nucleic Acids Res..

[28] Bairoch A., Apweiler R., Wu C.H., Barker W.C., Boeckmann B., Ferro S., Gasteiger E., Huang H., Lopez R., Magrane M. (2005). The universal protein resource (uniprot). Nucleic Acids Res..

[29] Hartigan J. (1981). Consistency of single linkage for high-density clusters. Am. Stat..

[30] Hastie T., Tibshirani R., Friedman J. (2009). The Elements of Statistical Learning.

[31] Hartigan J., Van Ryzin J. (1977). Distribution Problems in Clustering. Classification and Clustering.

[32] Barnett V. (1981). Interpreting Multivariate Data.

[33] Stanberry L., Nandy R., Cordes D. (2003). Cluster analysis of fMRI data using dendrogram sharpening. Hum. Brain Mapp..

[34] Murua A., Stanberry L., Stuetzle W. (2008). On Potts model clustering, kernel *K*-means, and density estimation. J. Comput. Graph. Stat..

[35] Stanford Center for Biomedical Informatics Research (BMIR) at the Stanford University School of Medicine Protege Project.

[36] Kamburov A., Pentchev K., Galicka H., Wierling C., Lehrach H., Herwig R. (2011). ConsensusPathDB: Toward a more complete picture of cell biology. Nucleic Acids Res..

[37] Bukreyev A., Whitehead S.S., Prussin C., Murphy B.R., Collins P.L. (2000). Effect of coexpression of interleukin-2 by recombinant respiratory syncytial virus on virus replication, immunogenicity, and production of other cytokines. J. Virol..

[38] Haynes L.M., Moore D.D., Kurt-Jones E.A., Finberg R.W., Anderson L.J., Tripp R.A. (2001). Involvement of toll-like receptor 4 in innate immunity to respiratory syncytial virus. J. Virol..

[39] Rallabhandi P., Phillips R.L., Boukhvalova M.S., Pletneva L.M., Shirey K.A., Gioannini T.L., Weiss J.P., Chow J.C., Hawkins L.D., Vogel S.N. (2012). Respiratory syncytial virus fusion protein-induced toll-like receptor 4 (TLR4) signaling is inhibited by the TLR4 antagonists Rhodobacter sphaeroides lipopolysaccharide and eritoran (E5564) and requires direct interaction with MD-2. mBio.

[40] Burgel P., Nadel J. (2004). Roles of epidermal growth factor receptor activation in epithelial cell repair and mucin production in airway epithelium. Thorax.

[41] Ornitz D.M., Itoh N. (2001). Fibroblast growth factors. Genome biology.

[42] Monick M.M., Cameron K., Staber J., Powers L.S., Yarovinsky T.O., Koland J.G., Hunninghake G.W. (2005). Activation of the epidermal growth factor receptor by respiratory syncytial virus results in increased inflammation and delayed apoptosis. J. Biol. Chem..

[43] Laplante M., Sabatini D.M. (2009). mTOR signaling at a glance. J. Cell Sci..

[44] DeFrancesco L. (2012). Omics gets personal. Nat. Biotechnol..

[45] Li-Pook-Than J., Snyder M. (2013). iPOP goes the world: Integrated personalized omics profiling and the road toward improved health care. Chem. Biol..

[46] Blumenberg M. (2012). SKINOMICS: Transcriptional profiling in dermatology and skin biology. Curr. Genomics.

[47] Gonzalez de Castro D., Clarke P.A., Al-Lazikani B., Workman P. (2013). Personalized cancer medicine: Molecular diagnostics, predictive biomarkers, and drug resistance. Clin. Pharmacol. Therapeutics.

[48] Pesce F., Pathan S., Schena F.P. (2013). From -omics to personalized medicine in nephrology: Integration is the key. Nephrol. Dial. Transpl. Off. Publ. Eur. Dial. Transpl. Assoc.-Eur. Renal Assoc..

[49] Rojo Venegas K., Aguilera Gmez M., Caada Garre M., Snchez A.G., Contreras-Ortega C., Calleja Hernndez M.A. (2012). Pharmacogenetics of osteoporosis: Towards novel theranostics for personalized medicine?. Omics J. Integr. Biol..

[50] Chen R., Snyder M. (2013). Promise of personalized omics to precision medicine. Wiley Interdiscip. Rev. Syst. Biol. Med..

[51] Mias G.I., Snyder M. (2013). Personal genomes, quantitative dynamic omics and personalized medicine. Quant. Biol..

[52] Zaas A.K., Chen M., Varkey J., Veldman T., Hero A.O., Lucas J., Huang Y., Turner R., Gilbert A., Lambkin-Williams R. (2009). Gene expression signatures diagnose influenza and other symptomatic respiratory viral infection in humans. Cell Host Microbe.

[53] Rosenberger C.M., Podyminogin R.L., Navarro G., Zhao G.W., Askovich P.S., Weiss M.J., Aderem A. (2012). miR-451 regulates dendritic cell cytokine responses to influenza infection. J. Immunol. (Baltimore, Md.: 1950).

[54] Swan M. (2013). The quantified self: Fundamental disruption in big data science and biological discovery. Big Data.

[55] Lanza G., Ferracin M., Gaf R., Veronese A., Spizzo R., Pichiorri F., Liu C.g., Calin G.A., Croce C.M., Negrini M. (2007). mRNA/microRNA gene expression profile in microsatellite unstable colorectal cancer. Mol. Cancer.

[56] Panguluri S.K., Bhatnagar S., Kumar A., McCarthy J.J., Srivastava A.K., Cooper N.G., Lundy R.F., Kumar A. (2010). Genomic profiling of messenger RNAs and microRNAs reveals potential mechanisms of TWEAK-induced skeletal muscle wasting in mice. PLoS One.

[57] Hrydziuszko O., Viant M.R. (2011). Missing values in mass spectrometry based metabolomics: An undervalued step in the data processing pipeline. Metabolomics.

[58] Webb-Robertson B.J.M., Matzke M.M., Metz T.O., McDermott J.E., Walker H., Rodland K.D., Pounds J.G., Waters K.M. (2013). Sequential projection pursuit principal component analysis-dealing with missing data associated with new -omics technologies. BioTechniques.

[59] Weckwerth W. (2007). Metabolomics: Methods and Protocols. Methods in Molecular Biology.

[60] Kolker E., Higdon R., Haynes W., Welch D., Broomall W., Lancet D., Stanberry L., Kolker N. (2012). MOPED: Model organism protein expression database. Nucleic Acids Res..

